# Treatment outcomes and factors affecting unsuccessful outcome among new pulmonary smear positive and negative tuberculosis patients in Anqing, China: a retrospective study

**DOI:** 10.1186/s12879-018-3019-7

**Published:** 2018-03-05

**Authors:** Yufeng Wen, Zhiping Zhang, Xianxiang Li, Dan Xia, Jun Ma, Yuanyuan Dong, Xinwei Zhang

**Affiliations:** 1 School of Laboratory Medicine, Wannan Medical College, 22 West Wenchang Road, Wuhu, Anhui Province 241002 People’s Republic of China; 2 Tuberculosis Prevention and Control Department, Anqing Center for Disease Control and Prevention, Anqing City, Anhui Province 246003 People’s Republic of China

**Keywords:** New pulmonary tuberculosis, Unsuccessful treatment outcomes, Retrospective study

## Abstract

**Background:**

Monitoring the treatment outcomes of tuberculosis and determining the specific factors associated with unsuccessful treatment outcome are essential to evaluate the effectiveness of tuberculosis control program. This study aimed to assess treatment outcomes and explore the factors associated with unsuccessful outcomes among new pulmonary smear positive and negative tuberculosis patients in Anqing, China.

**Methods:**

A nine-year retrospective study was conducted using data from Anqing Center for Diseases Prevention and Control. New pulmonary tuberculosis patients treated with two six-month regimens were investigated. Non-conditional logistic regression was performed to calculate odds ratios and 95% confidence intervals for factors associated with unsuccessful outcomes.

**Results:**

Among 22,998 registered patients (16,939 males, 6059 females), 64.54% were smear-positive patients. The treatment success rates was 95.02% for smear-positive patients and 95.00% for smear-negative patients. Characteristics associated with an higher risk of unsuccessful treatment among smear-positive patients included aged above 35 years, treatment management model of self-medication, full-course management and supervision in intensive phase, unchecked chest X-ray, cavity in chest X-ray, and miliary shadow in chest X-ray, while normal X-ray was negative factor. Unsuccessful treatment among smear-negative patients was significantly associated with age over 45 years, treatment management model of full-course management, unchecked chest X-ray, presence of miliary shadow in chest X-ray and delay over 51 days.

**Conclusions:**

Tuberculosis treatment in Anqing area was successful and independent of treatment regimens. Special efforts are required for patients with unsuccessful outcomes.

## Background

Tuberculosis (TB) remains a serious public health problem across the world. Treatment outcome is an important indicator of TB control programs. Patients failed the TB treatment are more likely to develop acquired drug resistant tuberculosis (DR-TB) [1]. Therefore, it is important to understand the factors associated with poor treatment outcome of TB and take appropriate measures.

Factors contributing to poor treatment outcome of TB are likely to vary depending on the local settings of populations. Studies among Chinese TB patients found that patients who were younger, lacking of cavitation, complying with treatment, supervised by health workers, living at study site, having higher income and receiving home visiting service from health workers had higher treatment success rates [2, 3]. Previous reports emphasized that in Ethiopia, factors independently associated with increased risk of unsuccessful TB treatment outcome were older age, habitation in rural areas, lack of contact person, sputum smear negative treatment category at initiation of treatment, smear positive sputum test result at second month after initiation treatment, retreatment cases and HIV positive status [4–6]. In a study in European area showed that the risk of unsuccessful treatment was significantly higher among male patients of foreign origin and increasing age, who were multidrug-resistant tuberculosis (MDR-TB) cases [7]. In addition, unemployment [8], longer duration of TB symptoms [9], lower educational level [10] and diabetes [11] were also risk factors for unsuccessful treatment outcome.

China is one of the world’s 22 countries with the highest burden of TB. There were an estimated 918,000 people infected with TB in China in 2015 [12]. In addition, 6.6% of new cases and 30% of previously treated cases were MDR-TB in 2015, much higher than the global level [12]. The treatment success rates varied significantly from 74.5% in Shandong Province [2], to 88% in Guangzhou [3] and up to 94.3% in north-west China [13]. However, treatment outcomes for TB patients in Anhui Province, a high TB incidence region, have not been evaluated so far.

Two types of 6-month treatment regimens are available for new smear-positive and new smear-negative TB patients in China. Regimen 1 (2H_3_R_3_Z_3_E_3_/4H_3_R_3_) is ethambutol (E), isoniazid (H), rifampicin (R) and pyrazinamide (Z) once-every-other-day for 2 months, followed by 4 months of once-every-other-day R and H. Regimen 2 (2HRZE/4HR) is daily E, H, R and Z for 2 months followed by 4 months of daily R and H. However, the impact of these two anti-TB regimens on treatment outcomes in Anhui Province has not been assessed.

Therefore, we conducted this study to assess the treatment outcomes of new pulmonary tuberculosis (PTB) patients and to explore factors associated with unsuccessful TB treatment outcomes among new pulmonary smear positive and negative tuberculosis patients in Anqing area, southwest Anhui Province.

## Methods

### Study area

Anqing area is situated at Southwest Anhui Province, covering 13.59 thousand square kilometers with a population of 5.32 million. In 2013, the rural population was 3.16 million and the per capita gross domestic product (GDP) was 26,657.89 RMB. Anqing area was selected not only for its high proportion of farming population and high TB prevalence but also because it is a typical rural area in Central China. Thus the results of this study can represent Central China to a significant extent.

### Study design and data collection

A retrospective study was conducted among new PTB patients who were registered at the Center for Diseases Prevention and Control (CDC) in Anqing city from 2005 to 2013. PTB patients were diagnosed on the basis of clinical manifestation, sputum smear and culture results, and radiological findings on chest X-ray. In our study, only new PTB cases aged 15 years-or-above who started standardized anti-TB treatment with regimen 1 (2H_3_R_3_Z_3_E_3_/4H_3_R_3_) and regimen 2 (2HRZE/4HR) directly after registration were selected for analysis. After excluding patients below 15 years old, retreatment patients, patients with extra-pulmonary tuberculosis (EPTB) and patients not treated with regimen 1 and regimen 2, the final sample size was 22,998.

Data were collected from the national TB epidemic Reporting System of Anqing CDC. The characteristics and clinical data of patients included: age, sex, ethnicity, occupation, bacteriology results, chest X-ray findings, ways of discovering patients, treatment management models, onset of symptom (such as cough, expectoration, etc.), information on the first care-seeking visit at any health system (i.e., health centers, hospitals or TB treatment centers), the initiation of anti-tuberculosis treatment and treatment outcomes.

### Operational definition

According to the standard definitions of the Guidelines for Implementing the National Tuberculosis Control Program in China (2008) [14] and WHO guideline [12], the following clinical case and treatment outcome operational terms were used:

1. New PTB patient. A patient never took anti-TB drugs, receiving irregular TB treatment for less than 1 month (excluding the use of anti-TB drugs due to other diseases).
2. Smear-positive PTB (PTB+). A patient with two positive direct smear microscopy results, or one positive direct smear microscopy result and one positive sputum culture for Mycobacterium tuberculosis (MTB), or one positive direct smear microscopy result and radiographic abnormalities consistent with active PTB as determined by a clinician.
3. Smear-negative PTB (PTB-). A patient with three negative sputum smear results, chest imaging showing lesions of active PTB and one of the following: (a) suspected PTB symptom as cough, expectoration and hemoptysis; (b) strongly positive purified protein derivative (PPD) reaction; (c) positive anti-MTB antibody response; (d) lesions of TB confirmed by histopathological examination of extra-pulmonary tissues. In addition, a patient with positive sputum culture for MTB but negative sputum smear result is also a PTB- case.
4. Treatment outcomes

According to WHO guideline [12], treatment outcomes were categorized into: successful outcome (cured and completed treatment) and unsuccessful outcome (death, failure, defaulted and transferred out).

#### Cured

PTB+ patients who have completed full course of treatment and have two consecutive negative smear results including one after completion of therapy.

#### Completed treatment

PTB- patients who completed the prescribed course of treatment and have a negative sputum smear microscopy result or do not receive smear examination after completion of therapy; and PTB+ patients who completed the prescribed course of treatment, and do not receive smear examination after completion of therapy, but but the latest sputum smear result was negative.

#### Death

PTB patients who died from any cause during treatment.

#### Failure

PTB+ patients with positive sputum smear or culture results at month five or later during treatment; and PTB- patients with conversion to sputum smear positive during treatment.

#### Defaulted

Patients whose treatments were interrupted for two consecutive months or more.

#### Transferred out

Patients whose treatment results were unknown due to transfer to another health facility.

(5) Total delay: Total delay was defined as the time interval between the onset of any tuberculosis symptom and the initiation of anti-TB treatment.

### Statistical analysis

IBM SPSS for Windows Version 18.0 was used for statistical analyses. Data are presented with frequencies and percentages or median and interquartile range for socio-demographic, clinical characteristics and treatment outcomes. The proportions were compared using Chi-square. To evaluate the potential predictor variables of unsuccessful treatment outcome, we compared socio-demographic and clinical variables between the successful and unsuccessful treatment outcome groups, using univariate and multivariate logistic regression model. Variables with a *P*-value < 0.20 in the univariate analysis were included in the multivariate logistic regression model. In constructing regression model procedures, stepwise backward selection methods based on wald test were used to select statistically significant variables for unsuccessful treatment outcome, and the goodness of fit and collinearity of the model were tested with Hosmer-Lemeshow and Tolerance methods (without giving the results). The final multivariate logistic regression model included all the indicators of the significant variables by using the enter option in SPSS. *P*-value < 0.05 was taken as indicative of statistically significant difference.

## Results

### General characteristics of the study population

There were 25,014 PTB patients registered in Anqing CDC between 2005 and 2013. Among the 22,998 new PTB patients included in our study, 64.54% (14,842/22998) were PTB+ patients, 73.65% were males, and the median age was 51 years (interquartile range: 35–64). Most patients were farmers (76.12%) and treated with 2H3R3Z3E3/4H3R3 (76.45%). A majority of patients had abnormal X-ray manifestations (97.60%), and only 13.96% and 0.73% patients had cavity and miliary shadow in X-ray, respectively. There were significant differences in sex, age, occupation, X-ray, cavity and treatment regimen between PTB+ and PTB- patients (Table 1).

**Table 1:** Socio-demographic and clinical characteristics of new pulmonary tuberculosis patients in Anqing, China, 2005–2013 (*n* = 22,998)

Variables		Total (*n* = 22,998)	PTB+ (*n* = 14,842)	PTB- (*n* = 8156)	*χ* ^*2*^	*P*
Sex	Female	6059 (26.35)	3793 (25.56)	2266 (27.78)	13.46	< 0.001
	Male	16,939 (73.65)	11,049 (74.44)	5890 (72.22)		
Age	15~ 24	3197 (13.90)	1960 (13.21)	1237 (15.17)	54.30	< 0.001
	25~ 34	2281 (9.92)	1567 (10.56)	714 (8.75)		
	35~ 44	3601 (15.66)	2412 (16.25)	1189 (14.58)		
	45~ 54	3615 (15.72)	2340 (15.77)	1275 (15.63)		
	55~ 65	5013(21.80)	3269 (22.03)	1744 (21.38)		
	≥65	5291 (23.01)	3294 (22.19)	1997 (24.49)		
Ethnicity	Han	22,960 (99.83)	14,819 (99.85)	8141 (99.82)	0.27	0.61
	Other	38 (0.17)	23 (0.15)	15 (0.18)		
Occupation	Student	1414 (6.15)	807 (5.44)	607 (7.44)	115.29	< 0.001
	Official staff	356 (1.55)	190 (1.28)	166 (2.04)		
	Service	437 (1.90)	277 (1.87)	160 (1.96)		
	Worker	1897 (8.25)	1214 (8.18)	683 (8.37)		
	Farmer	17,507 (76.12)	11,567 (77.93)	5940 (72.83)		
	Retired staff	433 (1.88)	261 (1.76)	172 (2.11)		
	Unemployment	338 (1.47)	198 (1.33)	140 (1.72)		
	Other	616 (2.68)	328 (2.21)	288 (3.53)		
X-Ray	Normal	408 (1.77)	286 (1.93)	122 (1.50)	14.35	< 0.001
	Abnormal	22,447 (97.60)	14,447 (97.34)	8000 (98.09)		
	Unchecked	143 (0.62)	109 (0.73)	34 (0.42)		
Cavity	Yes	3210 (13.96)	2527 (17.03)	683 (8.37)	328.07	< 0.001
	No	19,788 (86.04)	12,315 (82.97)	7473 (91.63)		
Miliary shadow	Yes	169 (0.73)	99 (0.67)	70 (0.86)	2.64	0.10
	No	22,829 (99.27)	14,743 (99.33)	8086 (99.14)		
Regimen	Regimen1^a^	17,581 (76.45)	12,470 (84.02)	5111 (62.67)	1332.81	< 0.001
	Regimen 2^b^	5417 (23.55)	2372 (15.98)	3045 (37.33)		

PTB+: Smear-positive pulmonary tuberculosis patients

PTB-: Smear-negative pulmonary tuberculosis patients

^a^2H3R3Z3E3/4H3R3. R = rifampicin; H = isoniazid; Z = pyrazinamide; E = ethambutol. It means once-every-other-day HRZE for 2 months followed by 4 months of once-every-other-day HR

^b^2HRZE/4HR. It means daily HRZE for 2 months followed by 4 months of daily HR.

### Outcomes of treatment of the patients

The treatment success rate was not significantly different between PTB+ and PTB- patients (95.02% vs. 95.00%, *P* = 0.94) (Table 2). The nine-year trend in treatment success rate of PTB patients was steady (94.16% to 96.20%). The cure rate decreased markedly from 88.23% in 2005 to 36.10% in 2013, while completed treatment increased dramatically from 6.38% in 2005 to 58.41% in 2013. Generally, the rates of failure, death and transfers did not follow a definite pattern, but they presented falling tendencies. Moreover, the rate of default rose from 0.25% in 2005 to 2.27% in 2013. In addition, regimen 2 was used to treat PTB in 2008 and widely used since 2011 (Table 3).

**Table 2:** Treatment outcomes of new pulmonary tuberculosis patients registered for treatment between 2005 and 2013, Anqing, China (*n* = 22,998)

Treatment outcome	Total (*N* = 22,998)	PTB+ (*N* = 14,842)	PTB- (*N* = 8156)	*χ* ^*2*^	*P*
Successful	21,851 (95.01)	14,103 (95.02)	7748 (95.00)	0.01	0.94
Cured	14,003 (60.89)	14,003 (94.35)	0		
Treatment completed	7848 (34.12)	100 (0.67)	7748 (95.00)		
Unsuccessful	1147 (4.99)	739 (4.98)	408 (5.00)		
Failure	126 (0.55)	108 (0.73)	18 (0.22)		
Death	157 (0.68)	111 (0.75)	46 (0.56)		
Default	330 (1.43)	189 (1.27)	141 (1.73)		
Transferred out	534 (2.32)	331 (2.23)	203 (2.49)		

PTB+: Smear-positive pulmonary tuberculosis patients

PTB-: Smear-negative pulmonary tuberculosis patients

**Table 3:** Trends of treatment outcomes, treatment regimens and pulmonary tuberculosis patients type of patients in Anqing, China, 2005–2013 (*n* = 22,998)

Variables	2005 (*n* = 1631)	2006 (*n* = 1576)	2007 (*n* = 1870)	2008 (*n* = 3267)	2009 (*n* = 3134)	2010 (*n* = 3499)	2011 (*n* = 2739)	2012 (*n* = 2861)	2013 (*n* = 2421)
Successful	1543 (94.60)	1484 (94.16)	1799 (96.20)	3111 (95.22)	2992 (95.47)	3307 (94.51)	2619 (95.62)	2708 (94.65)	2288 (94.51)
Cured	1439 (88.23)	1255 (79.63)	1248 (66.74)	2350 (71.93)	2107 (67.23)	2106 (60.19)	1572 (57.39)	1052 (36.77)	874 (36.10)
Treatment completed	104 (6.38)	229 (14.53)	551 (29.47)	761 (23.29)	885 (28.24)	1201 (34.32)	1047 (38.23)	1656 (57.88)	1414 (58.41)
Unsuccessful	88 (5.40)	92 (5.84)	71 (3.80)	156 (4.78)	142 (4.53)	192 (5.49)	120 (4.38)	153 (5.35)	133 (5.49)
Failure	18 (1.10)	12 (0.76)	6 (0.32)	12 (0.37)	20 (0.64)	26 (0.74)	6 (0.22)	10 (0.35)	16 (0.66)
Death	22 (1.35)	14 (0.89)	14 (0.75)	18 (0.55)	16 (0.51)	21 (0.60)	18 (0.66)	18 (0.63)	16 (0.66)
Default	4 (0.25)	10 (0.63)	7 (0.37)	41 (1.25)	38 (1.21)	70 (2.00)	47 (1.72)	58 (2.03)	55 (2.27)
Transferred out	44 (2.70)	56 (3.55)	44 (2.35)	85 (2.60)	68 (2.17)	75 (2.14)	49 (1.79)	67 (2.34)	46 (1.90)
Regimens									
Regimen 1^a^	1631	1576	1870	2864 (87.66)	3005 (95.88)	3448 (98.54)	1837 (67.07)	692 (24.19)	658 (27.18)
Regimen 2^b^	0	0	0	403 (12.34)	129 (4.12)	51 (1.46)	902 (32.93)	2169 (75.81)	1763 (72.82)
Patients type									
PTB+	1526 (93.56)	1348 (85.53)	1298 (69.41)	2488 (76.16)	2203 (70.29)	2252 (64.36)	1660 (60.61)	1128 (39.43)	939 (38.79)
PTB-	105 (6.44)	228 (14.47)	572 (30.59)	779 (23.84)	931 (29.71)	1247 (35.64)	1079 (39.39)	1733 (60.57)	1482 (61.21)

PTB+: Smear-positive pulmonary tuberculosis patients

PTB-: Smear-negative pulmonary tuberculosis patients

^a^2H3R3Z3E3/4H3R3. R = rifampicin; H = isoniazid; Z = pyrazinamide; E = ethambutol. It means once-every-other-day HRZE for 2 months followed by 4 months of once-every-other-day HR

^b^2HRZE/4HR. It means daily HRZE for 2 months followed by 4 months of daily HR

### Multivariate analysis to identify independent factors associated with unsuccessful outcome

Based on a multivariate analysis, factors found to be associated with unsuccessful treatment outcome among PTB+ patients included age groups of 35–44 years (Odds ratio [OR] = 1.56, 95% Confidence interval [CI]: 1.00–2.42, *P* = 0.049); 45–54 years (OR = 2.12, 95% CI:1.38–2.60, *P* < 0.001); 55–64 years (OR = 2.32, 95% CI: 1.52–3.53, *P* < 0.001) and 65 years-and-above (OR = 3.08, 95% CI: 2.03–4.68, *P* < 0.001). Others include treatment management model of self-medication (OR = 70.38, 95% CI: 12.97–381.85, *P* < 0.001), full-course management (OR = 16.45, 95% CI: 11.17–24.22, *P* < 0.001), and supervision in intensive phase (OR = 10.86, 95% CI: 7.91–14.91, *P* < 0.001); normal and unchecked chest X-ray (OR = 0.09, 95% CI: 0.01–0.63, *P* = 0.016 and OR = 2.70, 95%CI: 1.41–5.16, *P* = 0.003), cavity in chest X-ray (OR = 1.42, 95% CI: 1.18–1.73, *P* < 0.001), and miliary shadow in chest X-ray (OR = 3.39, 95% CI 1.87–6.13, *P* < 0.001) (Table 4).

**Table 4:** Factors associated with unsuccessful treatment outcomes among new pulmonary smear-positive tuberculosis patients registered during 2005–2013 in Anqing, China (*n* = 14,842)

Variables		Total (*n* = 14,842)	Unsuccessful (*n* = 739)	Univariate analysis		Multivariate analysis	
				OR (95% CI)	*P*	OR (95% CI)	*P*
Sex	Female	3793 (25.56)	177 (4.67)	1			
	Male	11,049 (74.44)	562 (5.09)	1.09 (0.92, 1.30)	0.30		
Age	15~ 24	1960 (13.21)	48 (2.45)	1		1	
	25~ 34	1567 (10.56)	49 (3.13)	1.29 (0.86, 1.93)	0.22	1.31 (0.81, 2.11)	0.28
	35~ 44	2412 (16.25)	87 (3.61)	1.49 (1.04, 2.13)	0.03	1.56 (1.00, 2.42)	0.049
	45~ 54	2340 (15.77)	113 (4.83)	2.02 (1.43, 2.85)	< 0.001	2.12 (1.38, 3.60)	< 0.001
	55~ 64	3269 (22.03)	182 (5.57)	2.35 (1.70, 3.24)	< 0.001	2.32 (1.52, 3.53)	< 0.001
	≥65	3294 (22.19)	260 (7.89)	3.41 (2.50, 4.67)	< 0.001	3.08 (2.03, 4.68)	< 0.001
Ethnicity	Han	14,819(99.85)	739 (4.99)	–	0.63		
	Other	23 (0.15)	0	1			
Occupation	Student	807 (5.44)	21 (2.60)	1			
	Official staff	190 (1.28)	6 (3.16)	1.22 (0.49, 3.07)	0.67		
	Service	277 (1.87)	9 (3.25)	1.26 (0.57, 2.78)	0.57		
	Worker	1214 (8.18)	54 (4.45)	1.74 (1.04, 2.91)	0.03		
	Farmer	11,567 (77.93)	618 (5.34)	2.11 (1.36, 3.28)	< 0.001		
	Retired staff	261 (1.76)	16 (6.13)	2.44 (1.26, 4.76)	0.009		
	Unemployment	198 (1.33)	7 (3.54)	1.37 (0.58, 3.27)	0.48		
	Other	328 (2.21)	8 (2.44)	0.94 (0.41, 2.13)	0.87		
Way of discovering patients	Health examination	112 (0.75)	4 (3.57)	1			
	Contact examination	12 (0.08)	1 (8.33)	2.46 (0.25, 23.94)	0.44		
	Clinic visit due to symptoms	6716 (45.25)	294 (4.38)	1.24 (0.45, 3.38)	0.68		
	Recommended due to symptoms	204 (1.37)	17 (8.33)	2.46 (0.81, 7.48)	0.11		
	Referral	7137 (48.09)	370 (5.18)	1.48 (0.54, 4.03)	0.45		
	Tracing	637 (4.29)	52 (8.16)	2.40 (0.85, 6.77)	0.10		
	Other	24 (0.16)	1 (4.17)	1.17 (0.13, 11.00)	0.89		
Treatment management models	Self-medication	8 (0.05)	6 (75.00)3	67.72(13.65, 336.07)	< 0.001	70.38 (12.97, 381.85)	< 0.001
	Full-course supervision	14,512 (97.78)	615 (4.24)	1		1	
	Full-course management	122 (0.82)	50 (40.98)	15.69(10.84, 22.71)	< 0.001	16.45 (11.17, 24.22)	< 0.001
	Supervision in intensive phase	200 (1.35)	68 (34.00)	11.64(8.59, 15.77)	< 0.001	10.86 (7.91, 14.91)	< 0.001
X-Ray	Normal	286 (1.93)	1 (0.35)	0.07(0.01, 0.47)	0.007	0.09 (0.01, 0.63)	0.016
	Abnormal	14,447 (97.34)	726 (5.03)	1		1	
	Unchecked	109 (0.73)	12 (11.01)	2.34(1.28, 4.28)	0.006	2. 70 (1.41, 5.16)	0.003
Cavity	Yes	2527 (17.03)	154 (6.09)	1.30 (1.08, 1.56)	0.005	1.42 (1.18, 1.73)	< 0.001
	No	12,315 (82.97)	585 (4.75)	1		1	
Miliary	Yes	99 (0.67)	14 (14.14)	3.18 (1.80, 5.63)	< 0.001	3.39 (1.87, 6.13)	< 0.001
	No	14,743 (99.33)	725 (4.92)	1		1	
Regimen	Regimen 1^a^	12,470 (84.02)	610 (4.89)	1			
	Regimen 2^b^	2372 (15.98)	129 (5.44)	1.12 (0.92, 1.36)	0.26		
Total delay	≤17	3965 (26.71)	181 (4.56)	1			
	17~ 33	3632 (24.47)	173 (4.76)	1.05 (0.85, 1.29)	0.68		
	33~ 66	3594 (24.22)	164 (4.56)	1.00 (0.81, 1.24)	> 0.99		
	> 66	3651 (24.60)	221 (6.05)	1.35 (1.10, 1.65)	0.004		

^a^2H3R3Z3E3/4H3R3. R = rifampicin; H = isoniazid; Z = pyrazinamide; E = ethambutol. It means once-every-other-day HRZE for 2 months followed by 4 months of once-every-other-day HR

^b^2HRZE/4HR. It means daily HRZE for 2 months followed by 4 months of daily HR

Factors associated with unsuccessful treatment outcome among PTB- patients were age groups of 45–54 years (OR = 1.79, 95% CI: 1.04–3.09, *P* = 0.04) and 65 years-and-above (OR = 2.57, 95% CI: 1.52–4.35, *P* < 0.001). others are treatment management model of full-course management (OR = 2.54, 95% CI: 1.70–3.81, *P* < 0.001), unchecked chest X-ray (OR = 3.77, 95% CI: 1.50–9.45, *P* = 0.005), miliary shadow in chest X-ray (OR = 2.43, 95% CI: 1.12–5.25, *P* = 0.02) and delay of over 51 days (OR = 1.40, 95% CI: 1.06–1.85, *P* = 0.019) (Table 5).

**Table 5:** Factors associated with unsuccessful treatment outcomes among new pulmonary smear-negative tuberculosis patients in Anqing, China, 2005–2013 (*n* = 8156)

Variables		Total (*n* = 8156)	Unsuccessful outcomes	Univariate analysis		Multivariate analysis	
				OR (95% CI)	*P*	OR (95% CI)	*P*
Sex	Female	2266 (27.78)	112 (4.94)	1			
	Male	5890 (72.22)	296 (5.03)	1.02 (0.81, 1.27)	0.88		
Age	15~ 24	1237 (15.17)	36 (2.91)	1		1	
	25~ 34	714 (8.75)	28 (3.92)	1.36 (0.82, 2.25)	0.23	1.34 (0.73, 2.47)	0.34
	35~ 44	1189 (14.58)	43 (3.62)	1.25 (0.80, 1.96)	0.33	1.22(0.69, 2.16)	0.50
	45~ 54	1275 (15.63)	65 (5.10)	1.79 (1.18, 2.71)	< 0.001	1.79 (1.04, 3.09)	0.04
	55~ 64	1744 (21.38)	87 (4.99)	1.75 (1.18, 2.60)	< 0.001	1.59 (0.93, 2.74)	0.09
	≥65	1997 (24.49)	149 (7.46)	2.69 (1.86, 3.90)	< 0.001	2.57 (1.52, 4.35)	< 0.001
Ethnicity	Han	8141 (99.82)	407 (5.00)	0.74 (0.10, 5.62)	0.54		
	Other	15 (0.18)	1 (6.67)	1			
Occupation	Student	607 (7.44)	19 (3.13)	1			
	Official staff	166 (2.04)	12 (7.23)	2.41 (1.15, 5.08)	0.02		
	Service	160 (1.96)	6 (3.75)	1.21 (0.47, 3.07)	0.69		
	Worker	683 (8.37)	28 (4.10)	1.32 (0.73, 2.39)	0.36		
	Farmer	5940 (72.83)	321 (5.40)	1.77 (1.10, 2.83)	0.02		
	Retired staff	172 (2.11)	13 (7.56)	2.53 (1.22, 5.23)	0.01		
	Unemployment	140 (1.72)	4 (2.86)	0.91 (0.31, 2.72)	0.87		
	Other	388 (3.53)	5 (1.74)	0.55 (0.20, 1.48)	0.23		
Way of discovering patients	Health examination	165 (2.02)	5 (3.03)	1			
	Contact examination	40 (0.49)	2 (5.00)	1.68 (0.32, 9.01)	0.54		
	Clinic visit due to symptoms	3548 (43.50)	175 (4.93)	1.66 (0.67, 4.10)	0.27		
	Recommended due to symptoms	30 (0.37)	1 (3.33)	1.10 (0.12, 9.79)	0.93		
	Referral	3793 (46.51)	197 (5.19)	1.75 (0.71, 4.32)	0.22		
	Tracing	559 (6.85)	27 (4.83)	1.62 (0.62, 4.29)	0.33		
	Other	21 (0.26)	1 (4.76)	1.60 (0.18, 14.39)	0.68		
Treatment management models	Self-medication	30 (0.37)	4 (13.33)	2.84 (0.96, 8.41)	0.06	2.95 (0.98, 8.90)	0.055
	Full-course supervision	1049 (12.86)	54 (5.15)	1		1	
	Full-course management	513 (6.29)	59(11.50)	2.40 (1.63, 3.52)	< 0.001	2.54 (1.70, 3.81)	< 0.001
	Supervision in intensive phase	6564 (80.48)	291 (4.43)	0.86 (0.63, 1.15)	0.30	0.95 (0.70, 1.30)	0.76
X-Ray	Normal	122 (1.50)	0	–	–	< 0.01 (< 0.01, > 999.99)	0.96
	Abnormal	8000 (98.09)	402 (5.03)	1		1	
	Unchecked	34 (0.42)	6 (17.65)	4.00(1.67–10.00)	0.002	3.77 (1.50, 9.45)	0.005
Cavity	Yes	683 (8.37)	32 (4.69)	0.93 (0.64, 1.34)	0.69		
	No	7473 (91.63)	376 (5.03)	1			
Miliary shadow	Yes	70 (0.86)	8 (11.43)	2.48 (1.18, 5.21)	0.02	2.43 (1.12, 5.25)	0.02
	No	8086 (99.14)	400 (4.95)	1		1	
Regimen	Regimen 1^a^	5111 (62.67)	278 (5.44)	1			
	Regimen 2^b^	3045 (37.33)	130 (4.27)	0.78 (0.63, 0.96)	0.02		
Total delay	≤13	2047 (25.10)	94 (4.59)	1		1	
	13~ 27	2054 (25.18)	80 (3.89)	0.84 (0.62, 1.14)	0.27	0.84 (0.62, 1.14)	0.27
	27~ 51	2046 (25.09)	100 (4.89)	1.07 (0.80, 1.43)	0.66	1.05(0.78, 1.41)	0.74
	> 51	2009 (24.63)	134 (6.67)	1.49 (1.13, 1.95)	0.004	1.40 (1.06, 1.85)	0.019

^a^2H3R3Z3E3/4H3R3. R = rifampicin; H = isoniazid; Z = pyrazinamide; E = ethambutol. It means once once-every-other-day HRZE for 2 months followed by 4 months of once-every-other-day HR

^b^2HRZE/4HR. It means daily HRZE for 2 months followed by 4 months of daily HR

## Discussion

In this retrospective study, we assessed treatment outcomes and the associated factors for unsuccessful treatment among new PTB patients between 2005 and 2013 in Anqing area. Most patients (73.65%) were males, which was consistent with other studies in Southern Ethiopia [5], Uzbekistan [9]and Penang, Malaysia [10]. This finding also follows the epidemiological trend of TB in China [12, 15]. The reasons for the higher rates in males may be due to the differential susceptibility to TB caused by biological mechanisms, lower notification in females caused by socioeconomic and cultural barriers in accessing health services and higher risk of exposure to TB through social interactions in males [16]. In addition, 44.81% new PTB patients were aged above 55 years. This is different from the data in studies in Uzbekistan [8] and Southern Ethiopia [5] where most patients were in the productive age group (15–55 years). However, older patients in our study might make TB control more complex and difficult, because they had lower treatment success rates than younger patients [17].

The treatment success rate was 95.01% in our study, which is consistent with summarized treatment success rate of 93.9% among new TB cases in the mainland of China [18] and data from studies elsewhere [13, 19–21]. Moreover, the treatment success rate obtained here is relatively higher than the values of 85.2% in southern Ethiopia [5]; 83% in Uzbekistan [8]; 78% in the European Union and European Economic Area [7] and 67.26% in Penang, Malaysia [10]. It is also higher than reported rates in other areas in China, in which treatment success rates ranging from 74.5% to 94.3% were reported [2, 3, 13, 22]. The comparably more successful treatment outcome reported in this study demonstrates the success and promising performance of TB control in the study area.

The unsuccessful treatment rate in this study was comparably lower than 14.8% in Southern Ethiopia [5] and 16.4% in Arsi Zone, Central Ethiopia [4]. The differences could be ascribed to the fact that we did not include EPTB patients and retreatment cases in our study. EPTB patients are more likely to have unsuccessful treatment outcome than PTB+ patients [5], and retreatment cases have much lower treatment success rate than PTB+ cases [4]. The transferred-out cases in this study comprised a major portion of the unsuccessful outcome, which is in agreement with findings in similar studies in western Ethiopia [23, 24]. Incidence of default, death and failure were significantly different from data from other researches in other parts of the world [5, 8, 10, 19, 24–26].

Trends in treatment success rate remained relatively stable over the nine-year period. However, the trend in default was significantly increased from 0.25% in 2005 to 2.27% in 2013. Therefore, specific measures are needed to improve treatment compliance among PTB patients in Anqing area. In addition, the proportion of PTB- patients increased over the period (from 6.44% in 2005 to 61.21% in 2013). Previous studies in San Francisco showed that a smear-negative TB patient contributed to 17% (95% CI: 12–24%) of TB transmission [27]. Thus more attention should be paid to PTB- patients and more efforts should be made to improve their treatment outcomes.

A deeper understanding of factors associated with unsuccessful treatment outcomes can lead to appreciation of appropriate interventions for reducing morbidity and mortality. The present study indicates that the risk of unsuccessful TB treatment increases with the increasing age among new TB patients (whether PTB+ or PTB-) aged above 45 years. This is supported by other reports [3, 4, 7, 28]. Unsuccessful treatment among older TB patients was mainly due to higher default rates and deaths [22]. Lefebvre et al. reported that advancing age was the most significant determinant of death among TB patients [29]. Furthermore, atypical clinical manifestations in older TB patients and other concomitant age-related diseases can affect the diagnosis of TB, leading to increased mortality among the elderly [30]. Therefore, specific strategies are needed to quickly address TB management among TB patients aged above 45 years in this study area.

An inverse relationship between treatment success and delay in diagnosis and treatment of TB was observed in this study. This can be ascribed to the more serious complications, higher mortality [31] and MDR-TB [7, 32] caused by delay in diagnosis and treatment of TB. Measures must be taken to encourage TB patients to seek medical help as early as possible.

In addition, PTB patients with cavitations were 0.69 times less likely to have treatment success outcome than patients without cavitations [3]. This was corroborated among new PTB+ patients in this study. This can be attributed to cavitation associated with higher baseline sputum mycobacterial load [33]. Heavy initial bacillary load is associated with delay in smear conversion at the end of the intensive phase of TB treatment, and delay in smear conversion is independently associated with treatment failure and death [34]. Based on the results of this study, patients are encouraged to have X-ray examination and to pay more attention to abnormal chest X-ray manifestation, especially those having cavitations and miliary shadows.

One additional finding of our study was that PTB patients in treatment management model of full-course supervision and supervision in intensive phase had higher probability of treatment success. This is consistent with the study in China [22], which indicated that absence of a treatment observer was associated with unsuccessful outcome. It is also in conformity with the findings of a study in Brazil, which indicated that patients who did not receive directly observed therapy were more likely to default from anti-TB treatment, die of TB and have unknown treatment outcomes [35]. Our findings suggest that the treatment management model of supervision in intensive phase should be recommended for both new PTB+ and PTB- patients. Furthermore, unlike in other studies [7, 28], we could not find associations between males and unsuccessful treatment. In addition, no association was found between treatment regimens and treatment outcomes, which is inconsistent with the study in Nigeria [28] where 2RHZE/6EH treatment was a predictor of unsuccessful outcome in HIV negative TB patients.

Several limitations of this study must be considered. First, this study only investigated new PTB patients treated with two main regimens, so it cannot represent all TB patients in Anqing. Secondly, this study was conducted retrospectively, based on administrative data, which missed many socioeconomic data of the TB patients, such as lower educational level and lower income, which were reported to be risk factors of unsuccessful outcome [2, 10]. Thirdly, the database does not contain information on other potential factors such as compliance with treatment [3], sputum smear test result at second month after initiation treatment [6], duration of symptoms before treatment [9], co-morbidity [11], HIV status [4] and drug resistance status [7, 11], all of which are known to be associated with TB treatment outcomes. Furthermore, there was no information on patients’ TB awareness, duration of the treatment, distance from the treatment centre, or medication side effect. These variables may also affect treatment outcomes. In addition, although there 2.32% TB patients were transferred out, but it maybe not a potential confounding factor to our results, because the rates were not significant different between two treatment regimens and two different TB patients types, and the trend of this rate is remained stable.

## Conclusions

The treatment outcome of new PTB patients in Anqing area was successful. Transfer-out cases were the major unsuccessful treatment outcomes of tuberculosis patients in the study area. The treatment success rate should be maintained, and measures must be taken to reduce barriers to the unsuccessful outcomes. Our study has provided useful insights on factors associated with unsuccessful treatment outcome. These specific populations should be monitored intensively.

## Abbreviations

CDC: Center for Diseases Prevention and Control
EMB: Ethambutol
GDP: Gross domestic product
HIV: Human Immunodeficiency Virus
INH: Isoniazid
LTBI: Latent tuberculosis infection
MDR-TB: Multidrug-resistant tuberculosis
MTB: Mycobacterium tuberculosis
PPD: Purified protein derivative
PTB: Pulmonary tuberculosis
PTB-: Smear-negative PTB
PTB+: Smear-positive PTB
PZA: Pyrazinamide
RMP: Rifampicin
TB: Tuberculosis
WHO: World Health Organization
